# Review on Biomedical Advances of Hybrid Nanocomposite Biopolymeric Materials

**DOI:** 10.3390/bioengineering10030279

**Published:** 2023-02-21

**Authors:** Abeer M. Alosaimi, Randa O. Alorabi, Dina F. Katowah, Zahrah T. Al-Thagafi, Eman S. Alsolami, Mahmoud A. Hussein, Mohammad Qutob, Mohd Rafatullah

**Affiliations:** 1 Department of Chemistry, Faculty of Science, Taif University, P.O. Box 11099, Taif 21944, Saudi Arabia; 2 Chemistry Department, Faculty of Science, Ibb University, Ibb 70270, Yemen; 3 Department of Chemistry, Faculty of Applied Science, Umm Al-Qura University, P.O. Box 16722, Makkah 21955, Saudi Arabia; 4 Chemistry Department, Faculty of Science, King Abdulaziz University, P.O. Box 80203, Jeddah 21589, Saudi Arabia; 5 Chemistry Department, Faculty of Science, Assiut University, Assiut 71516, Egypt; 6 Environmental Technology Division, School of Industrial Technology, Universiti Sains Malaysia, Penang 11800, Malaysia; 7 Green Biopolymer, Coatings & Packaging Cluster, School of Industrial Technology, Universiti Sains Malaysia, Penang 11800, Malaysia

**Keywords:** hybrid materials, biomaterials, biomedical applications, carbon-based materials, bio-nanocomposites

## Abstract

Hybrid materials are classified as one of the most highly important topics that have been of great interest to many researchers in recent decades. There are many species that can fall under this category, one of the most important of which contain biopolymeric materials as a matrix and are additionally reinforced by different types of carbon sources. Such materials are characterized by many diverse properties in a variety industrial and applied fields but especially in the field of biomedical applications. The biopolymeric materials that fall under this label are divided into natural biopolymers, which include chitosan, cellulose, and gelatin, and industrial or synthetic polymers, which include polycaprolactone, polyurethane, and conducting polymers of variable chemical structures. Furthermore, there are many types of carbon nanomaterials that are used as enhancers in the chemical synthesis of these materials as reinforcement agents, which include carbon nanotubes, graphene, and fullerene. This research investigates natural biopolymers, which can be composed of carbon materials, and the educational and medical applications that have been developed for them in recent years. These applications include tissue engineering, scaffold bones, and drug delivery systems.

## 1. Introduction

Polymers, which are relatively inexpensive and simple to create, are indeed an essential group of materials in today’s climate [1,2]. They are made up of multiple repeating units known as monomers, the sum of which specifies the degree of polymerization. Polymers are classified according to their starting point (natural or synthetic), chemical composition (organic or inorganic), category of monomer unit (homopolymers or copolymers), indices of degradation (chemical, biological, etc.), stability (e.g., thermal, mechanical, etc.), and applications [3,4]. Polymers have appeal as ingredients of composites used in biomedical applications due to their exceptional features. Polymeric biomaterials have been a multidisciplinary and interdisciplinary field for about 50 years, and the progression of this field is linked to advances in chemistry, medicine, physics, biology, and materials science. The fundamental purpose of researchers working in this field is to generate biomimetic polymer hybrid materials of varied compositions and sizes (i.e., nano-macroscale) that have features that are acceptable for a variety of applications within the biomedical field. Furthermore, they should have dynamic and tunable properties that closely resemble their biological analogues. Their interactivity, in distinct, is critical for improving performance, achieving the required attributes for their particular purposes, and maximizing their manufacture [5].

The huge category of substances that have been utilized for medical purposes, such as surgical sutures and implants, are biomedical biopolymers. This widespread use can be attributed not only to the variety of these substances, the versatility of their applications, and the reasonable cost of doing business with them but also to the adaptability of their chemical composition, which bestows on them distinctive chemical, biological, and physical properties. In spite of the fact that many advancements have been carried out in polymer research, no single polymer can achieve everything. In particular, their low mechanical strength is an issue in biomedical applications. This is the motivation for research toward novel, polymer-based biomaterials with a variety of functional groups. Synthetic biomaterials coming from natural biopolymers offer ideal structural properties: the biopolymer provides its unique characterization or intensive variable interactions, while the preparative constituent contributes responsiveness, structural integrity, and low cost. The self-assembly of synthetic building blocks, biomacromolecules, and particularly biopolymers of supramolecular and dynamic characters have all come up with distinguished bioinspired or biomimetic components [3,4,5]. The creation of biopolymers with a variety of structures, such as composites, networks, blends, and copolymers, enables access to a wide range of biomaterials that have desirable advances.

## 2. Biomedical Biopolymer Nanocomposites

The behaviors and functions of a biopolymeric material are mostly governed by its preparation systems and crystallinity, average molecular weight, and molar mass distribution, which can be effectively displayed under laboratory procedures. Biopolymers of functional groups, in which the biopolymer backbone carries reactive functional groups with at least one attached at the backbone or the main chain, are utilized as building blocks for more advanced developed frameworks, including biocompatible materials, hydrogels, scaffolds, nanoparticles, and bio-conjugated surfaces. Biopolymeric substances designed for biomedical applications must satisfy specific achievements if they are to be used in vivo; therefore, understanding the different degradation processes attributed to biofilm tissue or blood interactions is essential. Biopolymers that are biostable or physiologically inert can withstand both hydrolytic and oxidative environments as well as sterilization conditions such as dry heat, chemicals, steam, precipitation, ethylene oxide, and ionizing radiation; they can also be used in longer-term applications such as artificial organs. On the other hand, biodegradable biopolymeric materials serve a temporary purpose, degrading into small molecular compounds according to a tunable degradation rate. These degradation products are excreted and/or metabolized; the bulk of biodegradable biopolymers are appropriate for surgical sutures, tissue engineering scaffolds, and drug delivery [6]. Biomedical biopolymers degrade via oxidation, hydrolysis, and physical or enzymatic degradation. Additionally, each process differs from the kinetics point of view; the main constituents that determine the degradation process and the rate are the design of the material, its water uptake, and its biological habitat (including pH). Biopolymers containing ester or amide groups degrade primarily through hydrolysis or oxidation, altering the material’s physical properties and potentially causing swelling and subsequent biopolymer degeneration via hydrolysis, whereas polyether-containing biopolymers have higher stability and can withstand lifelong exposure in the body. The weight of molecules, mechanical properties, and monomer release are commonly used parameters for monitoring the degradation process [7,8]. Natural biopolymers offer appealing characteristics such as chemical inertness, abundant availability, low toxicity, biodegradability, biocompatibility and cell signaling ability [9]; however, the prepared biopolymers exhibit complementing qualities, including mechanical and physical strength as well as chemical and thermal stability. Compared with synthetic polymers, the organized structure of natural biopolymers allows for enhancements including tissue ingrowth and cell viability improvement. However, polymers prepared by synthetic procedures can be more easily produced into variable microstructure forms based on their higher sensitivity during processing to temperature, pH, infrared (IR) or ultraviolet (UV) radiation, and solvents. Furthermore, shape-memory composite biomaterials can be designed in accordance with different stimuli such as electric, magnetic, or electromagnetic fields. The next generation of smart biomaterials is anticipated to accomplish functional tissue regeneration and supramolecular self-assembly on the cellular level [5]. The value of biopolymer gel materials in medical applications, such as tissue engineering, is comparable to the importance of other types of materials. As a result of their capacity to form tissues that are analogues of those found in vivo and to keep normal cellular function intact, biopolymer gel materials have found widespread application in the field of cell encapsulation [10].

### 2.1. Natural Biopolymers

**Figure 1:**
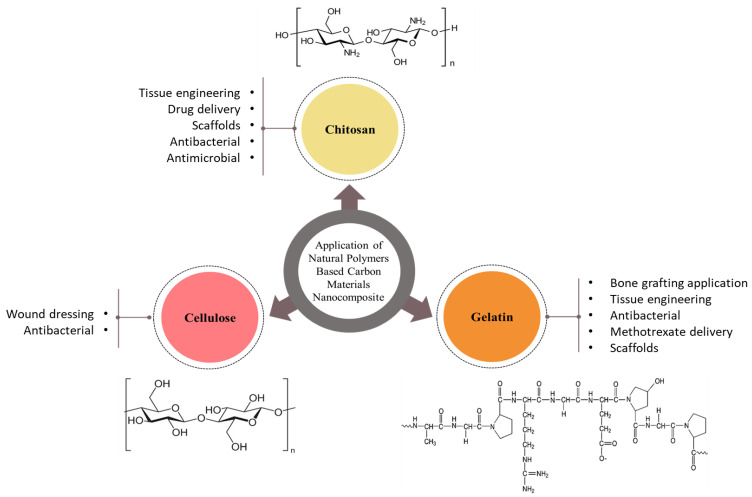
Schematic illustration of the most common natural biopolymers for biomedical applications.

Biopolymers from natural sources span a diverse range of structures with different physiological functions. Their properties are promising for biomedical applications and include high whiteness and crystallinity, high tensile strength and elasticity, high surface area [9], and, most importantly, biocompatibility and biodegradability. Furthermore, natural biopolymers contain many groups that can afford additional enzymatic type and/or chemical changes by somehow coupling with a variety of molecules, allowing for the synthesis of a huge variety of biomaterials with tailored structures and characters. Incorporating the natural biopolymers’ advantages with those of preparative types may allow for successfully mimicking the natural extracellular matrix (ECM) and obtaining biocomposites with predominant biological and mechanical performances [11,12]. In spite of that, most such natural biopolymers are sourced from vegetables, animals, worms, and spiders; many microorganisms are capable of synthesizing biopolymers. In addition, fish are major marine sources of biopolymers, along with corals, reptiles, algae, fungi, mammals, and marine invertebrates. Fish skin is a particularly rich source of collagen, while algae contain a variety of polysaccharides, and crustacean shells contain chitin and alginate [13]. The most common natural biopolymers for biomedical applications are depicted in Figure 1.

#### 2.1.1. Chitosan (CS)

CS is a polysaccharide from the linear type is generated from an *N*-deacetylation reaction with chitin, which is a natural polysaccharide as well [14]. CS is an amphiphilic natural copolymer that is suitable for forming hydrogel materials for biomedical applications. The use of CS as a hemostatic, fungi- and bacteriostatic agent, as well as in cell proliferation and tissue regeneration, is well known [15,16]. It can also be formed enzymatically by the chitinase enzyme or straight separated from fungi cell walls. The common commercial CS is usually available and fabricated via the fermentation of chitin, which is amenable to large-scale production. It was necessary to create derivatives with enhanced solubility and processability because CS is normally insoluble in neutral organic solvents due to the presence of acetyl, amino, and hydroxyl groups in the polysaccharide chain that establishes hydrogen bonds, making the chitin extremely aggregated. As a cation, CS forms interactions with negatively charged molecules such as proteoglycans and glycosaminoglycans, allowing it to act as a carrier for growth factors or cytokines.

CS possesses antimicrobial performances (antibacterial and antifungal), which can be additionally enhanced by chemical modifications, irradiation, partial hydrolyzation processes, and the application of antimicrobial agents, among other methods. Owing to its versatility, biocompatibility, biodegradability, low toxicity, and non-antigenic characteristics, CS is an appealing material for tissue engineering, drug delivery, and antimicrobial films, for example, in the tissue engineering field. In one study, the skins of pigs were regenerated with CS, and the results were strikingly identical to those of a patient’s own skin [17]. However, despite its many desirable properties, CS has low mechanical strength. Hence, the blending fabrication process is highly appreciated with variable ceramic sources or different polymers, including hydroxyapatite, for practical applications. CS forms colloidal particles and can produce bioactive encapsulated phenomena via either complex formation or chemical and ionic cross-linking to achieve controlled drug release. Other materials can also be combined with CS to form hydrogels, fibers, granules, or sponges [5].

#### 2.1.2. Gelatin (GL)

GL is a natural biopolymer obtained from collagen-containing animal tissue through hydrolysis and is used in various applications such as cosmetics, medical devices, and tissue engineering [18,19,20] due to its commercial availability, biodegradability, non-immunogenicity, biocompatibility, and cell interactivity [20,21,22,23,24]. GL is effective as a wound dressing material, absorbing exudates, providing moisture, and accelerating wound healing; however, it cannot prevent wound infection. The major component of drug-delivery capsules, GL can absorb between 5 and 10 times its mass in water. GL expedites the absorption of nutrients, drugs, and bioactive compounds by the body [25,26]. The arginine-glycine-asparagine (RGD)-like sequence found in GL also encourages cell adhesion and migration [27].

#### 2.1.3. Cellulose

A polysaccharide found in plants, cellulose is based on glucose moieties as repeating units. It closely resembles cellulose, lignin and many other polysaccharides with hydroxyl groups on their surfaces. Such a huge number of groups can easily promote the possibility of hydrogen bonds of both types (inter- and intramolecular). In addition, cellulose has the advantages of biodegradability, chirality, hydrophilicity, and a broad chemical-modifying capacity. A major drawback inhibiting the incorporation of cellulose nanofibers into composites is their incompatibility with hydrophobic biopolymers. Carboxymethyl cellulose was one of the first examples of modified cellulose materials used in the field of biomedicine. Several different chemical modification procedures have been tried out on cellulose in an effort to overcome this disadvantage. Some bacteria also release cellulose fibers extracellularly. *Acetobacter xylinum* (or *Gluconacetobacterxylinus*) is classified as a highly effective bacteria for this purpose, as it generates a more hydrated and pure cellulose membrane, eliminating the need for further chemical treatments to degrade lignin and hemicelluloses. Bacterial cellulose and plant cellulose have identical molecular formulas, but the former has a more complex 3D porous structure and is of higher purity and crystallinity. Bacterial cellulose also has high water content, great mechanical stability, and a high degree of polymerization and tensile strength, making it quite different from the plant form. In addition, bacterial cellulose is biocompatible, biodegradable, and moldable in the wet state, enhancing its appeal for applications in biomedicine. Moreover, it lacks both antioxidant and antibacterial characteristics, limiting its practical use. Composites composed of bacterial cellulose contain antioxidant, antifungal, antibacterial, and antiviral properties due to the use of bioactive biopolymers such as chitosan, polyethylene glycol, gelatin, and collagen. Biomaterials containing bacterial cellulose have been used in dental implants, skin replacements, bandages for wounds, blood vascular grafts, and burn treatments, in addition to inorganic particles such as silver, hydroxyapatite, montmorillonite, and biopolymeric nanoparticles [28].

### 2.2. Common Carbon Nano-Fillers

#### 2.2.1. Graphene

Graphene is a carbon-containing material with a 2-dimensional framework that has received a lot of awareness since its invention in 2004 by Geim and colleagues. Graphene is made up of a one-atom-thick planar sheet of sp2 carbon atoms in a honeycomb crystal lattice display. This structure is combined with strong in-plane C-C bonding, an aromatic form, and the existence of free p- and interacting sites for surface reactions, resulting in a special material with good mechanical, physicochemical, thermal, electrical, optical, and biological characteristics [29,30]. Many of its material parameters, including mechanical stiffness, strength, elasticity, and electrical and thermal conductivity, are superior [31]. Graphene is the most powerful and stretchable material known, and it is completely impermeable. It also has the highest thermal conductivity ever measured and extremely high fundamental mobility. Graphene possesses a number of characteristics that make it prospectively appealing for bio-applications. It has a large surface area, large chemical clarity, and ease of functionalization, all of which produce an excellent candidate for drug delivery, and its unique mechanical characteristics allow it to alter suitable tissue engineering performances. Functionalized graphene by chemical modifications may also find use in rapid, ultrasensitive display systems because it can identify various biological molecules such as glucose, cholesterol, hemoglobin, and DNA. Graphene is additionally lipophilic, which may aid in membrane barrier penetration, which is another challenge in drug delivery. Graphene and its nanocomposites have recently seen widespread use in biomedicine for drug/gene delivery, cancer therapy, tissue engineering, and bio-sensing [32].

#### 2.2.2. Carbon Nanotubes (CNTs)

CNTs are rounded graphene sheets that are seamless and have exceptional physical, mechanical, and chemical capabilities [33,34,35,36]. Based on the value of graphene (G) layers that make up a single nanotube, CNTs have been divided into two primary classes: single-walled carbon nanotubes (SWNTs) and multi-walled carbon nanotubes (MWNTs). CNTs have numerous applications, including as nanocomposite materials [37], nanoelectronics [38,39], field-effect emitters [40], and in hydrogen storage [41]. Due to their intriguing shape and size as well as their alluring and distinctive physical properties, efforts have been made over the past few years to look into the ideal biological applications of carbon nanotubes [42,43,44,45]. CNTs are more biocompatible than other carbon materials because they have fast electron-transfer kinetics, are ultra-lightweight and chemically inert, have high tensile strength, display a variety of antimicrobial characteristics (antibacterial and antifungal), function as protein transporters, and have reactive functional groups. Furthermore, CNTs are semi-metallic and metallic conductive, making them more elegant for clinical diagnostics, food treatment, and environmental monitoring. CNTs are furthermore used in cancer treatment and play an important role in the processing tools of sensors as an important part of variable pathogenic bacteria detection. A large number of CNTs are even antimicrobial in nature [46,47].

#### 2.2.3. Fullerenes

Numerous distinct functionalized fullerenes and nanocarbons display promise in biomedical applications, including in drug delivery, the quenching of reactive oxygen moieties, targeted imaging, and tissue engineering as a result of their superior chemical and physical characteristics [48,49,50,51,52,53,54,55]. Additionally, fullerenes’ photosensitivity makes them useful for photodynamic therapy and fighting multidrug-resistant pathogens [56,57]. Fullerenes have been reported to have biological antioxidant potential, with the ability to localize within mitochondria and other cell compartments where free radicals are produced [58]. Because fullerenes have a high number of conjugated double bonds and low-lying lowest unoccupied molecular orbitals (LUMOs) that can accommodate an electron, radical species can easily attack them. Fullerenes interact easily with a desirable number of superoxides and are non-consumable in the process, making them the best radical scavengers. Fullerenes and their derivatives may be antiviral due to their biological characteristics, such as their distinct genetic architecture and antioxidant activity, making them promising for treating infections, such as human immunodeficiency virus (HIV) [59].

## 3. Examples of Biomedical Applications

### 3.1. Natural Biopolymer/Carbon Nanocomposites

Because of its biocompatibility, antibacterial activity, and biodegradability, CS is an appealing biopolymer for the fabrication of scaffolds targeted for bone tissue engineering. Karimi et al. created CS-reinforced hydroxyapatite (HA)-graphene oxide nanocomposite coatings (GO) [60]. The CS/GO/HA coating was electrophoretically deposited onto a Ti substrate to improve osseointegration. The coating thickness, pore size, and water contact angle were all reduced as the CS loading was enlarged. An investigation into the effect of CS concentration on cell behavior revealed an increase in MG63 cell proliferation and viability from day 1 to day 7. The results also showed that the coating’s wettability and bonding strength were improved. An in vitro cytological analysis revealed that GO/CS/HA-Ti has superior bioactivity in bone marrow-derived mesenchymal stem cell adhesion, proliferation, and differentiation (BMSCs). Furthermore, in an in vivo animal study, this GO/CS/HA-Ti implant demonstrated superior osseointegration. Hydrophilic functional groups in GO, including carboxyl, carbonyl, and hydroxyl groups, have been shown in some studies to promote cell adhesion and proliferation.

The important characteristics, including the electrical, biological, and mechanical properties, of novel functionalized MWCNT/CS/β-glycerophosphate scaffolds have been investigated. The overall results show that composites with f-MWCNT are the most suitable for bone tissue engineering applications and cell electrical applications [61]. Mohamed and El-Ghany synthesized a number of modified CS derivatives [62]. In addition, two ratios of MWCNT biocomposites based on four were created. According to the findings, they had greater activity toward Gram-positive bacteria compared to Gram-negative bacteria. Additionally, a considerable inhibitory impact on the fungi tested has been demonstrated. By spoiling the microbial cell membrane and screening microbial growth, MWCNTs appeared to demonstrate a synergistic effect in the bacterial inhibition procedure. As a result, such derivatives and MWCNT hybrid materials are suitable for medical applications. F. Jabbari et al. compiled CS/silk fibroin (SF) and rGO membranes in a nanocomposite form with varying CS/SF/rGO weight ratios using a casting tool [63]. They then studied the physical, mechanical, and biological characteristics of these membranes to see if they could be used to regenerate bone tissue. In a biological study, human foreskin fibroblast cells were used. Red alizarin staining and alkaline phosphatase activity were used to assess osteogenic differentiation. The results revealed that as SF content increases, hydrophilicity, swelling, and degradability decrease, while tensile strength increases. Based on these findings, it is possible to conclude that blended membranes in the form of SF/CS/rGO are viable options for bone tissue engineering; an SF/CS/rGO membrane with a weight ratio of 84:7:9 was tested successfully for this purpose. More research should be done to tailor these membranes for other clinical applications. An easy and cost-effective reduction chemical procedure can be utilized to create new anticancer drug-loaded Pd nanoparticles (NPs) on CS/rGO. The presence of Pd nanospheres on CS/rGO promotes continuous drug release [64]. A cytotoxicity study on human colon cancer cells (HT-29) displayed that the framework can significantly inhibit cancer cell growth. Dhanavel et al. [65] studied the cytotoxicity of nanocomposites containing 5-fluorouracil (5-FU), curcumin (CUR), and dual-drug-loaded CS/rGO on HT-29 cancer cells. The semi-crystalline behavior of CS was displayed for all composites. The 5-FU and CUR peaks, as well as the CS/rGO reflection in the X-ray diffraction patterns, confirmed the successful loading of drugs into the nanocarrier. They discovered that HT-29 cells were toxic in a concentration-dependent manner. The superior inhibitory effect of dual-loaded drug composites on HT-29 cells suggested that the CS/rGO material is a viable transporter for multiple cancer therapeutic drugs. Saeednia et al. [66] created a thermosensitive, injectable CS hydrogel with G nanoparticles. They discovered that adding G to the CS hydrogels reduced the cumulative release of methotrexate (MTX), allowing for a slower and more controlled release of the drug. MCF-7 breast cancer cell growth was inhibited by nanohybrid hydrogels charged with MTX. Jiang et al. [67] created electrically conductive scaffolds with a porous structure made of CS blended with GO for cardiac tissue engineering. They came to the conclusion that the GO/CS scaffolds aided in cardiac tissue reconstruction. These findings provide information for the direction of new cardiac tissue engineering scaffolds. Moré et al. [68] created new films and scaffolds from low-molecular-weight chitosan (L-CS) and medium-molecular-weight chitosan (M-CS), resulting in composites with GO and apatite via a simple in situ self-assembly process that involved the direct partial reduction of GO by CS and the physical integration of apatite into the biopolymeric matrix. They also tested these composites’ ability to promote neural and fibroblast cell growth. They discovered that composites made from these materials are biocompatible and can be used as biomaterials in a variety of tissue regeneration applications. Another study loaded the drug famotidine (FMT) onto GO and encapsulated it with CS; the FMT was shown to chemically interact with GO [69]. FMT’s encapsulation performance was found to be best at an FMT:CSGO ratio of 1:9. This study is significant because this formulation of FMT exhibits long-term release activity with minimal clinical intervention and does not require repeated dosing.

Doxorubicin (DOX) is a chemotherapeutic agent that has a strong antitumor effect. Khoee et al. [70] showed that pH-responsive, CS-coated-GO mesoporous silica nanoparticles (GMSN-Cs) can be used as DOX delivery systems in cells. They investigated the impact of pH on drug liberation and discovered that the liberation of DOX was significantly greater at low pH values than in physiological conditions. Based on this discovery, the liberation of DOX-loaded GMSN-Cs can be striped by a physiological stimulant in order to minimize side impacts. Organic-inorganic nanocomposite materials made of Ag/graphite oxide (GtO)/CS and Ag/GO/CS have been used to create novel antibacterial nanomaterials [71]. The findings investigated that the nanocomposites had broad-spectrum antibacterial activity when applied to the two types of tested bacteria. The chitosan layer had no effect on the freeing of Ag from the nanocomposites, indicating their prospective utilization as biocidal compounds.

The assembly of chitosan-graphene in porous carbon resulted in an abundance of microneedles (MNs) [72]. The chitosan-carbon framework has high conductivity, which is advantageous for drug release induced by electric fields. The formulation also has antimicrobial properties, with CS, in conjunction with cephalexin, improving the antimicrobial activity of the nanocomposite. A biologically safe drug encapsulation model system for intelligent drug delivery was created using the microneedle approach (pH-dependent and electrical-field-triggered). A straightforward, useful technique for making CS/GO nanocomposite films has been applied for usage in orthopedic applications and bone tissue replacement [73]. A set of composite films were created using two different percentages of GO and four different percentages of CS as encouragement. The potential of the CS/GO nanocomposite for orthopedic bone replacement is shown by the improvement of the composite’s mechanical properties, thermal stability, bioactivity, and biodegradability with the addition of GO nanoparticles.

Cellulose seems to have desirable characteristics, including biocompatibility, hydrophilicity, biodegradability, and moisture retention, making it suitable for a variety of biomedical applications. A nanofiber composite of cellulose acetate/carbon nanotubes/silver nanoparticles (CA/CNT/Ag) was developed and found to be particularly suitable for antibacterial applications [74]. The CNT/Ag nanocomposites were fabricated by oxidizing the CNTs to create more -COOH and -OH groups and then electrospinning the CNT/Ag nanocomposites into the CA nanofibers. The antibacterial test results showed that the CA/CNT/Ag material has eminent antibacterial performance due to the production of reactive oxygen species, including peroxide ions, oxygen radicals, and hydroxyl ions, which rupture bacteria cell walls and damage their DNA, resulting in death and/or growth inhibition. According to the findings, CA/CNT/Ag nanocomposite materials are suitable materials for long-term antibacterial applications.

Yahia et al. [75] developed hybrid GO/HA/cellulose composite membranes with silver nanoparticles (AgNPs). This hybrid nanocomposite demonstrated effective antimicrobial activity while releasing only a small amount of Ag ions. More notably, the existence of GO improved AgNP deposition on cellulose fibers and prevented Ag ion leaching. The derived composites illustrated powerful antibacterial activity against both Gram-positive (*S. aureus*) and Gram-negative (*E. coli* and *Candida albicans*) bacteria, indicating that Ag/GO/HA has a strong antibacterial effect on cellulose. Ali et al. synthesized rGO from GO and then combined this rGO with a carboxymethyl cellulose-sodium salt (NaCMC) biopolymer to create a hydrogel. In comparison, the reduced form of GO was effective at removing biofilm [76]. The combined effect of rGO with NaCMC biopolymers to form rGO hydrogels, on the other hand, demonstrated a synergistic effect with the considerable inhibition (p.05) of biofilm at a very low concentration. Because of its ability to inhibit intrinsic virulence factors produced by bacteria, such as biofilms, this new rGO hydrogel may have the ability to restrict bacterial opposition. As a result, this rGO hydrogel formulation offers a powerful approach to the treatment of biofilm-infected wounds.

**Figure 2:**
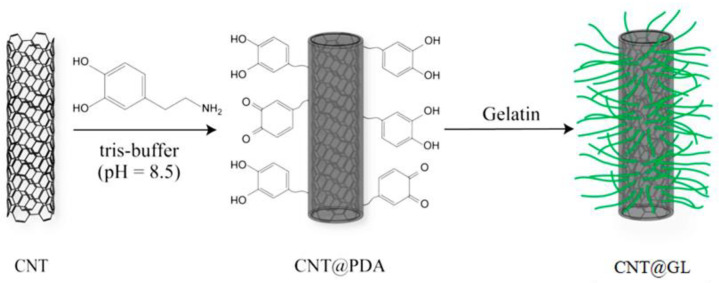
Illusion of the surface modification of CNT with GL [77].

One of the natural biopolymers commonly derived from the thermal hydrolysis of collagen is gelatin. It also has some additional characteristics, including biocompatibility, biodegradability, and hydrophilicity. As a result of its rheological and viscoelastic characteristics, GL is widely used throughout the fabrication of tissue engineering scaffolds for bone, cartilage, blood vessels, skin, tendons, and ligaments. Li et al. used a mussel-inspired method to treat CNT and coat it with poly(dopamine) using the abbreviation (PDA), and then they coated GL on the surface of CNT@PDA to form GL-modified CNT (CNT@GL), as shown in Figure 2 [77]. The in vitro test revealed that CNT@GL has significantly improved biocompatibility and cell viability over CNT alone, potentially facilitating its biomedical applications.

To continue improving the in vitro bioactivity of carbon–carbon composites (CC), functionalized composite coatings of CNT/GL/Zn-doped HA (FIC-GL-ZnHA) were produced using a hybrid method involving chemical vapor deposition (CVD), soaking, and electrochemical deposition (ECD) [78]. The FIC-GL-ZnHA coating, in particle form, was able to induce the formation of bone-like apatite. As a result, the FIC-GL-ZnHA-coated CC composite is a promising alternative to bone grafting. By filling a porous GL structure with GO aerogel, Zeinali et al. [79] created a hybrid scaffold for use in nerve tissue engineering. The hybrid scaffold exhibited a significantly enhanced elastic modulus, which is an indication that the hybrid scaffold was successful in its role as a strengthening agent. P19 mouse cells were cultured on the scaffold and distinguished into nerve cells for in vitro testing, which was followed by immunofluorescence testing. P19 cells were effectively identified into neural cells by the hybrid scaffold, making them viable candidates for nerve tissue engineering. Mesgar et al. [80] showed a freeze-dried GL/CS scaffold used for bone graft substitution in functionally adjusted multi-walled carbon nanotubes (f-MWCNTs). After freeze-drying the mixture, the structural, morphological, physicochemical, and comprehensive mechanical properties of two distinct types of G/CS scaffolds were investigated. The weight ratios used were 2:1 and 3:1, respectively. The formation of a bone-like apatite layer on the surface of scaffolds upon immersion in simulated body fluid was proof that the incorporation of f-MWCNTs improved in vitro bioactivity. This bone-like apatite layer allowed the reinforced f-MWCNT GL/CS scaffolds to be useful in bone tissue engineering. Arsalania et al. [81] developed a green and simple method for producing passive fluorescent carbon dots using GL and polyethylene glycol (PEG) as starting materials (CDs-PEG). This process produces high-quantum-yield (QY), carbon-dependent quantum dots from GL with great success. The authors looked into the effect of PEG on PL strength and discovered that CDs-PEG are more efficient than pristine CDs derived from GL. The anticancer capability of the CDs-PEG was assessed using in vitro tests, including the MTT assay and fluorescence microscopy. It demonstrated greater antitumor efficacy compared to the free MTX as a nanocarrier for the anticancer drug methotrexate (MTX) due to improved in vitro nuclear delivery, resulting in highly efficient tumor growth inhibition. As a result, CDs-PEG has the possibility to be used in targeted cancer therapy.

### 3.2. Hybrid Nanocomposites

Due to their amazing characteristics, including high mechanical strength, high surface area, high electrical conductivity, biocompatibility, and antibacterial properties, rGO nanosheets are also emerging as promising for tissue engineering. As a potential method of wound management, Khalili et al. created a biomaterial based on poly(phenylene sulfide) (PPS)/rGO/CS composite [82]. A hydrogel’s swelling properties are important in wound exudate adsorption; the CS swelling rate decreased from 800% to 200%. Cellular activity was attributed to the functional groups on the surface of rGO, which contributed to cell attachment in the samples. Overall, this PPS/rGO composite is a biomaterial that could be used in tissue engineering. CS/polyvinyl alcohol (PVA)/GO/HA/Au films were created for bone tissue regeneration, beginning with the hydrothermal preparation of GO/HA/Au nanocomposites [83]. The chemical stabilization and mechanical characteristics of the nanocomposite products showed excellent performance. The antibacterial activity and hemocompatibility of the CS/PVA/GO/HA/Au film were found to be high, and the results confirmed an improvement in osteoblast cell viability. As a result, these films can be used effectively for applications in bone tissue regeneration.

**Figure 3:**
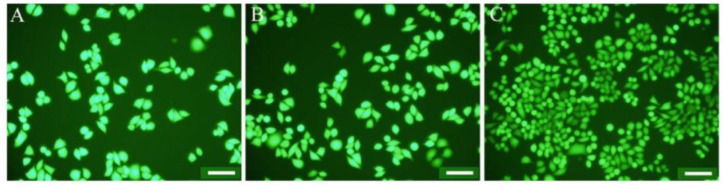
Photographs of L929 fibroblast cells incubated for 3 days on PCL fibers (**A**), PCL/CNT fibers (**B**), and CS-PCL/CNT fibers (**C**). Note: scale bars are 100 µm [84].

Another study used chitosan, a natural biopolymer, to develop the surface of electrospun polycabrolactone (PCL) fibers. The implementation of CNTs resulted in the preparation of negatively charged PCL fibers, which improved the mechanical strength of electrospun PCL fibers. The chitosan-modified composite demonstrated the highest antimicrobial performance versus *E. coli* and *S. aureus*. Furthermore, cell culture results revealed that the illustrated fibers’ composition was biocompatible and improved cell attachment and distribution onto the fibers. Figure 3 depicts the superior adhesion ability of living cells on the CS-PCL/CNT fiber mat compared to PCL fibers and PCL/CNT fibers. According to these findings, CNT incorporation improves CS immobilization onto electrospun PCL fibers. As a result, these composite materials are potentially useful biomaterials [84].

Since it is non-toxic, biodegradable, and biocompatible, CS is widely regarded as a superior antimicrobial product packaging. For example, Liu et al. [85] used electrospinning for the first time to create polylactic acid (PLA)/CNT/CS composite fibers for strawberry preservation. The exploratory results showed that increasing the CS content in PLA/MWCNT fibers improved the physical properties before degrading at a CS content of 7 wt percent. Furthermore, the composite fibers outperformed *E. coli*, *S. aureus*, *B. cinerea*, and *Rhizopus* in antimicrobial activity. The protection tests demonstrated that the nanocomposite fibers effectively preserved strawberries. As a result, the PLA/CNTs/CS composite fibers can slow physiological changes in strawberries and extend their shelf life, implying that they could be used to preserve other foods. Nano-microstructures are, however, suitable materials for tissue healing. As a result, nano-microhybrid scaffolds must be biodegradable, biocompatible, porous, have good mechanical properties, have 3D structures, and be similar to normal human tissue. A small amount of filler, such as MWNTs, improves the material’s properties. Electrospinning was used to create poly (3-hydroxybutyrate) and CS nano-microscaffolds that were then coated on a knitted silk microfiber substrate [86]. When compared to the scaffold without MWNTs, adding 1 wt percent MWNTs resulted in an improvement in features. The presence of COOH groups related to MWNTs is a good indicator of the scaffold’s bioactivity. The results showed that the scaffold containing MWNTs degraded at a slower rate, making it suitable for use in long-term applications such as cartilage tissue engineering

**Figure 4:**
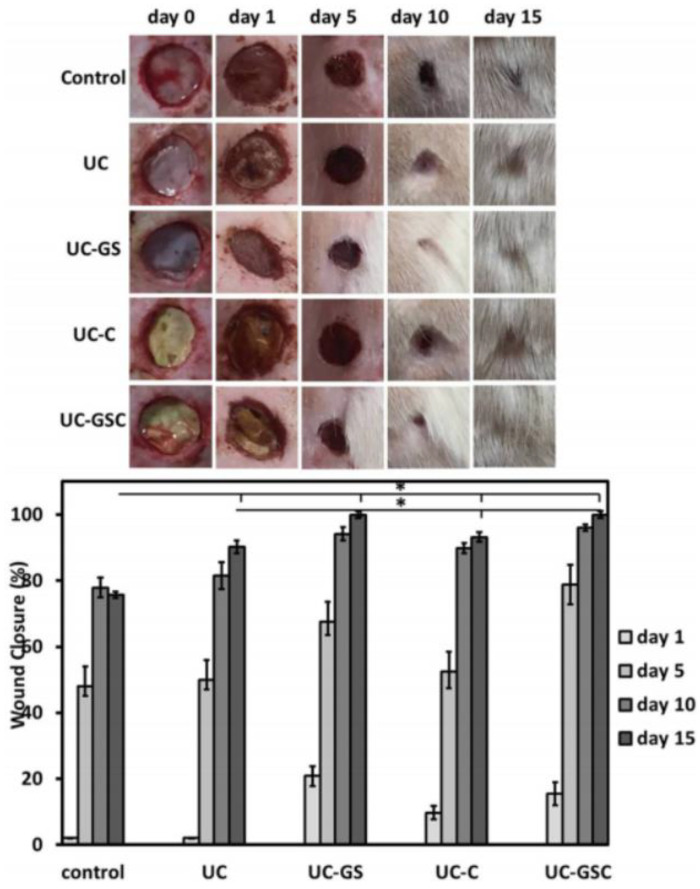
Visual observation and quantitative evaluation of healing wounds treated with the polyurethane/cellulose acetate named UC, where the UC contained rGO/Ag nanocomposites; UC-GS, where the UC contained curcumin; UC-C, where the UC contained rGO/Ag nanocomposites; and curcumin, UC-GSC [88]. *: These two samples were tested uder the same conditions and showed a fast healing rate compared to the others.

Currently, research is exploring the potential of nanocomposites containing biodegradable biopolymers such as PLA. A solvent casting method was used to create PLA reinforced with modified cellulose nanocrystals (CNC) and rGO [87]. The mechanical properties of the film with the highest wt percent of modified CNC were greatly improved. Furthermore, the nanocomposite film exhibited reduced water vapor barrier properties, antioxidant activity, induced biodegradability, and cytocompatibility, all of which are essential for health and packaging applications. Biopolymer nanocomposites have found widespread application in cutaneous wound healing as a result of recent advances in nanotechnology. Nanofibrous mats containing rGO/Ag and curcumin, for example, were created by electrospinning PU and cellulose acetate together [88]. The samples of rGO/Ag nanocomposites inhibited bacterial growth in both Gram-negative and Gram-positive bacteria. Reduced GO sheets act as a support for bacteria to adhere to due to their large surface area, while the release of Ag ions can destroy the bacteria’s membrane, which results in bacterial death. In vivo histopathological studies have revealed that the rGO/Ag scaffold with added curcumin had a powerful effect on wound healing and epidermis layer regeneration, including a faster adnexal healing response, as shown in Figure 4.

Hasani, Montazer, and colleagues [89] developed and modified CL/polyamide conductive fabric using rGO. Their antibacterial properties, high electrical resistance, and UV-protected fabric could be mass-produced for industrial, commercial, and military applications. In addition, using both reducing agents, the addition of cetyltrimethyl ammonium bromide allowed for higher loading of GO onto the tissue with enhanced electrical resistance and antimicrobial activities. By applying MWNTs to Poly (3-hydroxybutyrate) (PHB)/CS electrospun scaffolds, Karbasi et al. [90] examined the effects of MWNTs on the structural and the mechanical characteristics of PHB/CS electrospun scaffolds for use in cartilage tissue engineering. All of their findings revealed that 1 wt percent MWNTs is the best biopolymer solution concentration, with values comparable to cartilage. The mechanical as well as structural characteristics of the PHB-CS/MWNT scaffolds were adequate for utilization in cartilage tissue engineering [91]. MWNTs, along with chitosan and silk, provide an appropriate environment for the attachment and growth of chondrocytes. The P3HB-chitosan-MWNTs/silk (S) nano-microscaffold can be appropriate for long-term tissue engineering applications, such as cartilage. Another study demonstrated that PCL/GL/MWNTs might be considered a viable candidate for further tissue engineering research. Electrospinning was used to create PCL/GL scaffolds with various ratios of MWNTs [91]. The scaffolds containing MWNTs were also more bioactive and degraded at a slower rate. The nanocomposite scaffolds were found to be non-cytotoxic in cell cultures. This nanocomposite scaffold of PCL/GL/MWNTs has the potential to be used in cartilage tissue engineering applications. A simple solution extrusion process was used to create conductive sodium alginate/polyaniline (PANI)/graphene (PAG) neuron guidance channels [92]. The results showed that by incorporating the fabricated PAG composite material, the enhanced conductivity of the scaffolds expanded while the biodegradability of the conduit scaffolds decreased, which can be considered a perfect accomplishment given the hydrogel scaffolds’ high biodegradability. SEM analysis revealed that increasing the PAG loading led to a significant loss in the pore diameter of the scaffolds. Heshmatpour and Haghbin [93] created nano-HA/GO (nHA/GO) composites from natural CS as well as synthetic PEG and PVP and tested their biocompatibility. This was the first comprehensive investigation into the bioactivity of PEG and PVP-fabricated biopolymers in an nHA/GO hybrid material. All of the produced nHA/GO nanocomposites supplemented with synthetic polymers displayed cell proliferation and elevated alkaline phosphatase activity in MG-63 osteoblastic cells, in accordance with the findings. The nanocomposites also demonstrated significant bioactivity, comparable to traditional composites made of natural biopolymers. A pre-stressed double-network (DN) hydrogel nanocomposite was created using three primary materials: O-carboxymethyl CS, PVA, honey, and GO [94]. The GO was employed as a strengthening nanofiller in the matrix, serving as a physical cross-linker and being highly effective for cell implantation. GO contents of 3, 5, and 10% wt were investigated, and higher GO contents demonstrated some cytotoxicity. As a result of their antimicrobial, chemical, physical, and nanotopographic characteristics, GO and CS nanofibers have piqued the interest of researchers as wound dressings. Yang et al. [95] created a novel antimicrobial wound dressing by combining GO with nanofibrous CS/L-polylactic acid (PLLA) scaffolds. The GO coatings enhanced the surface roughness and water uptake of the nanofibrous CS/PLLA scaffolds while preserving the nanofiber shape and size. The antimicrobial activities of the GO-modified nanofibrous CS/PLLA against *S. aureus* and *E. coli* were greatly enhanced, and cell proliferation was promoted. All of the results showed that the GO-modified nanofibrous CS/PLLA scaffolds have a good potential for in vivo wound healing.

## 4. Conclusions

For the purpose of fabricating various forms of nanocomposites for use in biomedical applications, carbon-based nanoparticles of varying compositions have been used as fillers in natural biopolymeric materials. Several studies have been analyzed, and the findings have shown that natural biopolymer matrices such as chitosan, cellulose, and gelatin were used to reinforce carbon nanomaterials such as graphene, graphene oxide, reduced graphene oxide, carbon-based nanotubes, and fullerene to create bionanocomposites. These bionanocomposites were fabricated using a variety of techniques, including casting, dissolution, dispersion, and in situ methods. The various forms of bionanocomposites all showed considerable biologically active products against the bacteria, fungus, and in vitro cell cultures that were evaluated. However, from a future viewpoint, we need further research to keep pace with improvements in science and technology. This study has addressed a wide range of advancements in natural biopolymers, such as carbon nanomaterials and their applications in biomedicine.
